# Supernumerary human hair cells—signs of regeneration or impaired development? A field emission scanning electron microscopy study

**DOI:** 10.1080/03009734.2016.1271843

**Published:** 2017-02-01

**Authors:** Helge Rask-Andersen, Hao Li, Hubert Löwenheim, Marcus Müller, Kristian Pfaller, Annelies Schrott-Fischer, Rudolf Glueckert

**Affiliations:** a Department of Surgical Sciences, Head and Neck Surgery, Section of Otolaryngology, Uppsala University Hospital, Uppsala, Sweden;; b Department of Otolaryngology, Uppsala University Hospital, Uppsala, Sweden;; c Department of Otolaryngology, Medical University of Innsbruck, Innsbruck, Austria;; d Medical Campus University of Oldenburg School of Medicine and Health Sciences, European Medical School, Oldenburg, Germany;; e Research Center of Neurosensory Science, University of Oldenburg, Oldenburg, Germany;; f Cluster of Excellence Hearing4all, University of Oldenburg, Oldenburg, Germany;; g Department of Histology and Molecular Cell Biology, Institute of Anatomy and Histology, Medical University of Innsbruck, Innsbruck, Austria

**Keywords:** Human cochlea, inner hair cell, regeneration, SEM, supernumerary hair cells

## Abstract

**Background:**

Current attempts to regenerate cochlear sensorineural structures motivate further inspection of the human organ of hearing. Here, we analyzed the supernumerary inner hair cell (sIHC), a possible sign of regeneration and cell replacement.

**Methods:**

Human cochleae were studied using field emission scanning electron microscopy (FESEM; maximum resolution 2 nm) obtained from individuals aged 44, 48, and 58 years with normal sensorineural pure-tone average (PTA) thresholds (PTA <20 dB). The wasted tissue was harvested during trans-cochlear approaches and immediately fixed for ultrastructural analysis.

**Results:**

All specimens exhibited sIHCs at all turns except at the extreme lower basal turn. In one specimen, it was possible to image and count the inner hair cells (IHCs) along the cochlea representing the 0.2 kHz–8 kHz region according to the Greenwood place/frequency scale. In a region with 2,321 IHCs, there were 120 scattered one-cell losses or ‘gaps’ (5%). Forty-two sIHCs were present facing the modiolus. Thirty-eight percent of the sIHCs were located near a ‘gap’ in the IHC row (±6 IHCs).

**Conclusions:**

The prevalence of ectopic inner hair cells was higher than expected. The morphology and placement could reflect a certain ongoing regeneration. Further molecular studies are needed to verify if the regenerative capacity of the human auditory periphery might have been underestimated.

## Introduction

In 1884, the Swedish anatomist Gustav Retzius presented surface preparations of the human auditory epithelium (1). Lim and Lane (2) and Bredberg et al. (3) were the first to reveal the fine surface structure of the mammalian organ of Corti (OC) using scanning electron microscopy (SEM). This was followed by high-resolution SEM studies in humans (4–15). Electron microscopy studies of autopsied material are often limited by postmortem autolysis and age-related changes, and, to overcome this, perilymph fixation may be accomplished within hours after death.

**Figure 1.**
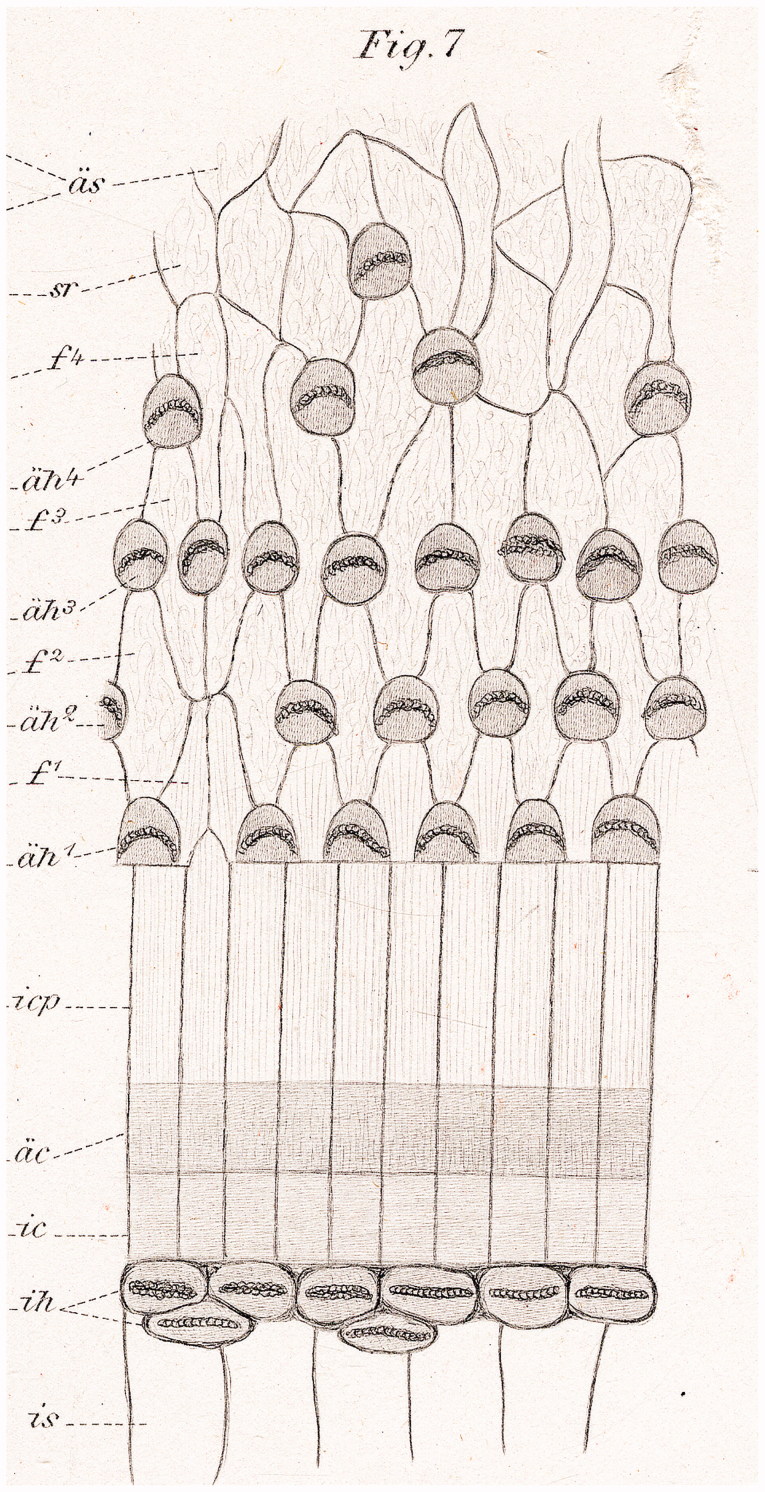
Surface pattern of the human cochlea (from Retzius 1884) (1).

Here, we used field emission scanning electron microscopy (FESEM) to analyze immediately fixed human cochleae removed at surgery. FESEM provides a maximum resolution of approximately 2 nm (16,17). Specimens were examined to investigate the fine structure and distribution of the so-called ‘extra’ or ‘supernumerary’ inner hair cells (sIHCs). Retzius (1) described sIHCs in the apical part of the mature rabbit cochlea and in the apical and middle turn of newborn humans (Figure 1). Since then, several authors have described sIHCs in various species (humans, rabbits, mouse, and rat) and speculated about their function (8,10,18–20). Ectopic or sIHCs appear during cochlear development, and there have been speculations that they may reflect an ongoing regeneration (21).

## Materials and methods

Three human cochleae were obtained during trans-cochlear surgery. During surgery, the facial nerve was re-routed postero-inferiorly and a petrosectomy performed. Instead of drilling through the cochlea, it was removed. The cochleae were put directly in fixative and transferred to the laboratory. The study was conducted in conformity with the Declaration of Helsinki principles, all patients provided informed consent, and the Ethics Committee of Uppsala University Hospital approved the study (No. 99398, 22/9 1999, 29/12 2013).

### Patient 1

Patient 1 (female, aged 48 years) exhibited extensive growth of a right-sided dermoid cyst (5 × 3.5 × 2 cm), which compressed the eighth cranial nerve and caused right-sided paralysis of the abducens nerve. Pure-tone audiometry was normal, with a speech discrimination of 72% on the right side. The cochlea was immediately fixed in 2.5% buffered glutaraldehyde for 7 days after removal. Decalcification was omitted; instead, the bony capsule was drilled away.

### Patient 2

Patient 2 (female, aged 58 years) suffered from a large, right-sided petro-clival meningioma. Audiometry was normal. After removal, the cochlea was fixed in 2.5% buffered glutaraldehyde and decalcified in 0.1 M Na-EDTA for 4 weeks.

### Patient 3

Patient 3 (female, aged 44 years) was operated on to remove a squamous cell carcinoma originating from the right external auditory meatus. A surgical labyrinthectomy was performed for radicality. Preoperative hearing thresholds showed a conduction hearing loss due to invasion of the tumor into the middle ear. Sensorineural functions were normal. After removal, the cochlea was immediately fixed in 2.5% buffered glutaraldehyde for 7 days.

### Field emission scanning electron microscopy (FESEM)

The specimens were dissected under an Olympus SZX9 stereomicroscope, washed in phosphate-buffered saline (pH 7.4) and dehydrated in a graded ethanol series, and critical-point dried using a CP Dryer (Balzers, Lichtenstein). They were attached to aluminum stubs using carbon glue (Planocarbon, Groepl, Austria), coated with a 10–15-nm layer of gold-palladium in a Baltech MED 020 coating system, and observed under a Zeiss DSM 982 Gemini field emission electron microscope operating at 4–5 kV. Maximum resolution was estimated to be 2 nm. Digital photographs were captured at a resolution of 1,280 × 1,024 pixels and stored in TIFF format. In specimen 1, the surface of the OC was photographed using overlapping exposures at 1,000× magnification such that inner hair cells could be counted and analyzed. A photomontage was constructed from serial digital photographs using Photoshop software, and inner hair cells (IHCs), sIHCs, and ‘gaps’ between the IHCs were counted. The lamina reticularis was analyzed for scarring at areas with missing hair cells.

## Results

### The human cochlea—a histological ‘challenge’

**Figure 2.**
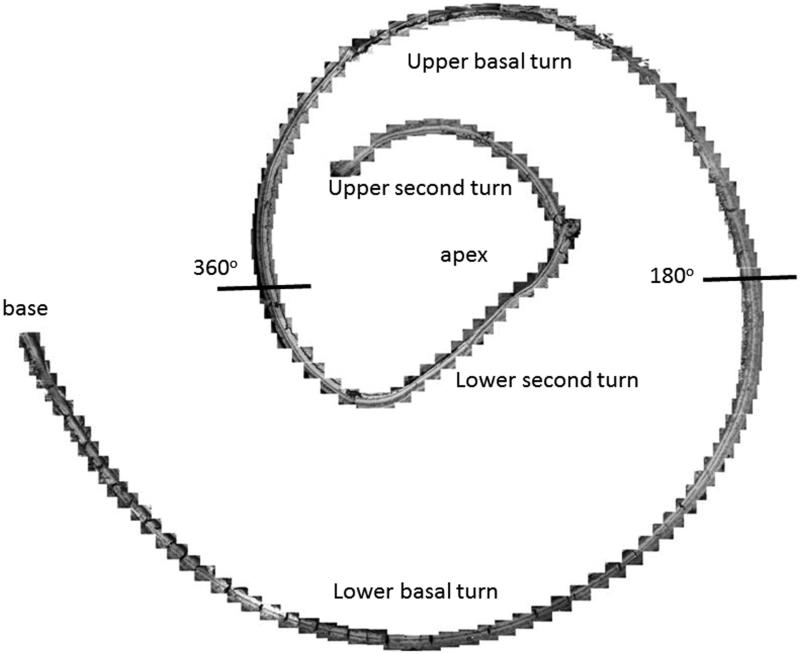
Composite SEM micrographs of the organ of Corti (specimen 1).

**Figure 3.**
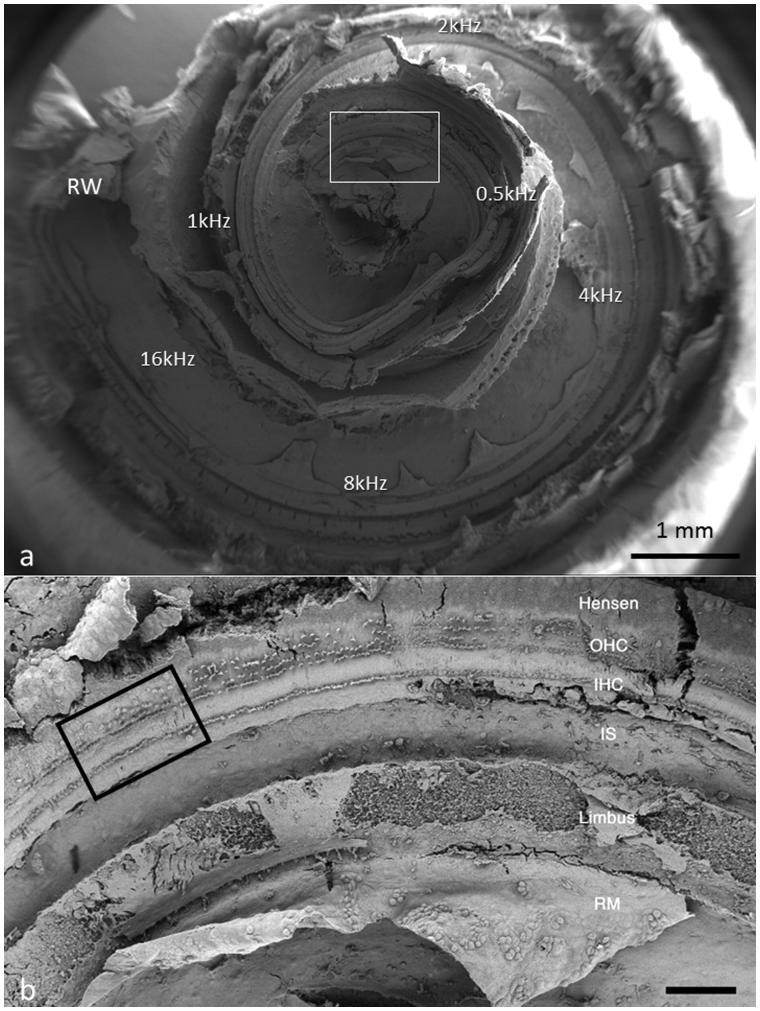
Image a: Field emission scanning electron microscopy (FESEM) of the cochlea of patient 1 (♀ 48 y.o.) with a normal audiogram (PTA). The framed area is magnified in image b. The most apical part was traumatized during preparation and is not shown. The IHCs were plotted and are shown in Figure 5. According to the Greenwood place/frequency scale, 2,321 IHCs were represented in the 0.2 kHz–4 kHz region (20,175 μm), corresponding to one hair cell per 8.7 μm or 11–12 IHCs per 100 μm of length. Image b: Higher magnification of the framed area shown in Image a. The exposed region corresponds to the junction between the upper second and lower apical turns of the cochlea.

**Figure 4.**
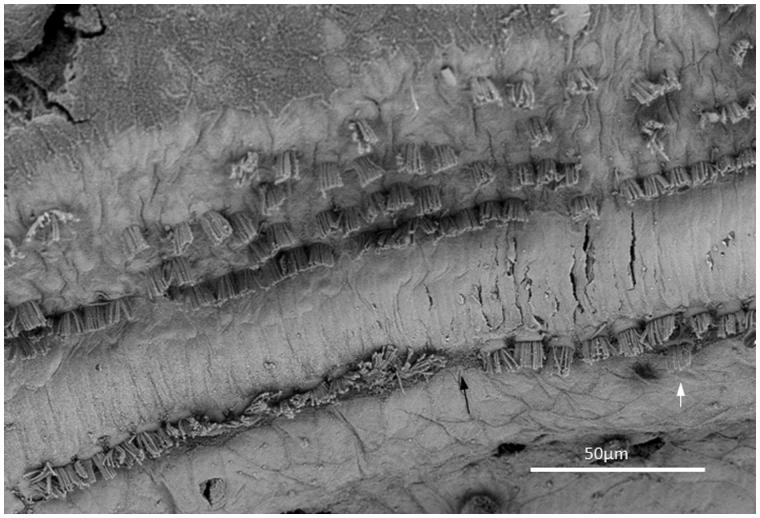
FESEM of the framed area shown in Figure 3(b). The image approximately represents the 300-Hz region. Four to five rows of OHCs are present, of which the second to fifth rows are incomplete. The left arrow shows one missing IHC. The right arrow shows a sIHC located against the modiolar side.

FESEM processing, including long-time Na-EDTA decalcification, preserved the surface structures and hair cells well in all specimens and allowed structure-audiometric correlations (Figures 2, 3, 4, 7, and 8). Diamond drilling on un-decalcified cochlea caused bone dust contamination and was further avoided. Two specimens could therefore not be used for quantitative assessment. The IHCs were surprisingly intact in these individuals, with few losses.

**Figure 5.**
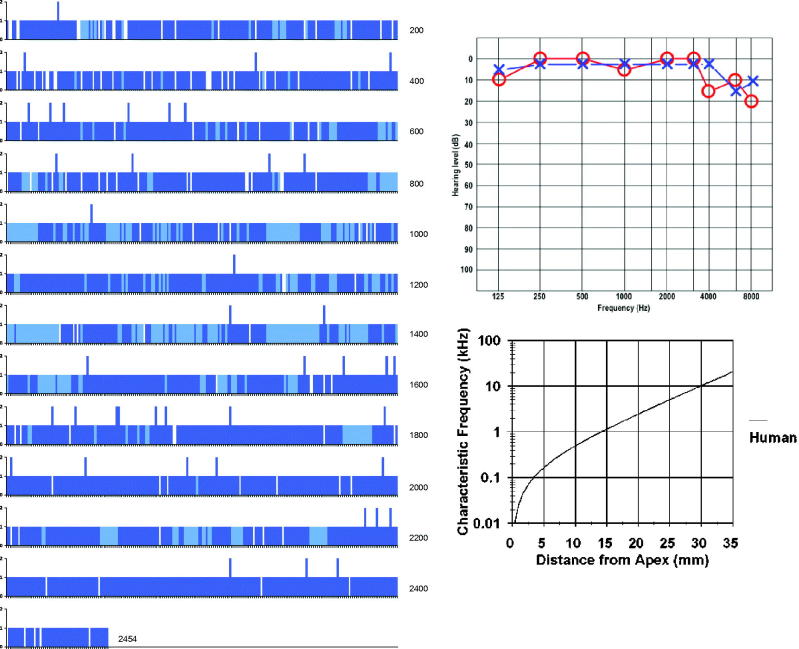
Missing IHCs (white bars) and sIHCs (blue bars). Light blue areas depict regions that are difficult to evaluate. The Greenwood place/frequency scale shows the characteristic frequencies (Hz) as the distance from the apex.

Different from most animals, the human hair cells are not strictly aligned in straight rows. In patient 1, it was possible to count all IHCs over a distance of 20,175 μm, which corresponded to the 0.2 kHz–8 kHz region according to the Greenwood place/frequency scale (Figures 2–5). Extrapolating the number of IHCs per unit length to the entire OC (35 mm) yielded a calculated total of 4,027 IHCs, a somewhat higher number than previously described (22). There were 2,321 IHCs, representing one hair cell per 8.7 μm or 11–12 IHCs per 100 μm of length. It contained 120 (5%) scattered IHC losses (mostly one cell wide) (Figure 5).

### Structure–audiometric correlations

**Figure 6.**
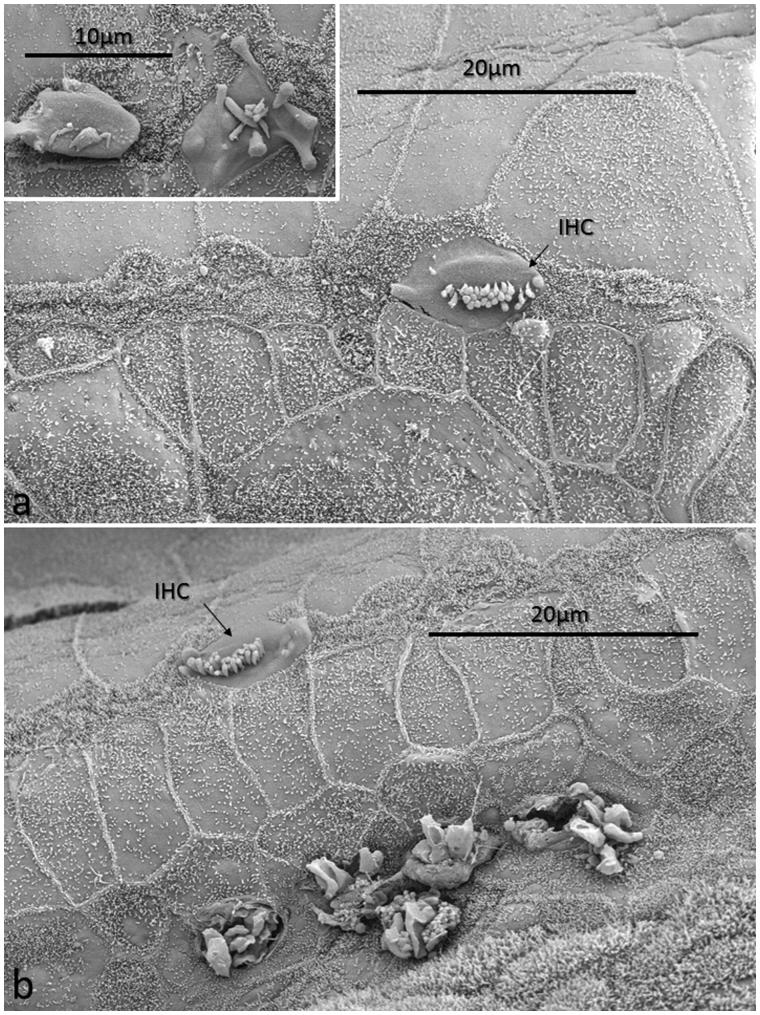
FESEM of the ‘hook’ region of the human cochlea. This region was generally devoid of hair cells. Image a: There is a solitary IHC with short stereocilia. Scarring of the reticular lamina is present. The inset shows two degenerated IHCs. Image b: A solitary IHC can be observed. The inner pillar heads appear preserved, whereas degenerated OHCs are observed below. Reparative processes or sIHCs are not apparent (patient 1).

The hair cell loss resulted in no recordable hearing loss at audiometry. In the ‘hook’ region, most hair cells were absent, and there was scarring of the lamina reticularis (Figure 6). At another place, three IHCs were degenerating, with the center cell missing (Figure 8(e–g)). In the basal turn, there were three rows of outer hair cells (OHCs), while in the apical region a fourth and fifth incomplete row of OHCs were recognized (Figure 3). OHCs were frequently missing but without obvious scarring, suggesting that it represented a biological irregularity rather than pathology. Some OHCs and IHCs showed giant cilia. One inner pillar cell head displayed ectopic stereocilia (not shown). IHCs had approximately 40–60 cilia per cell, arranged in three to four almost linear rows, with the longest cilia positioned laterally. Rudimentary kinocilia were seen at the lateral cell surface (Figure 8(c)). The cilia were not always arranged parallel to the hair cell row. Their width was estimated at 280–300 nm, as measured at mid-length. The short cilia were thinner than the long cilia. The distal tips of the long cilia were sometimes flat, whereas the short cilia often had pointed tips. At the base, the cilia narrowed into an anchoring rootlet. No inter-cilia links were observed, and cilia in the same row often exhibited different lengths. A few IHCs showed giant cilia.

### Ectopic inner hair cells—surprisingly common in humans

**Figure 7.**
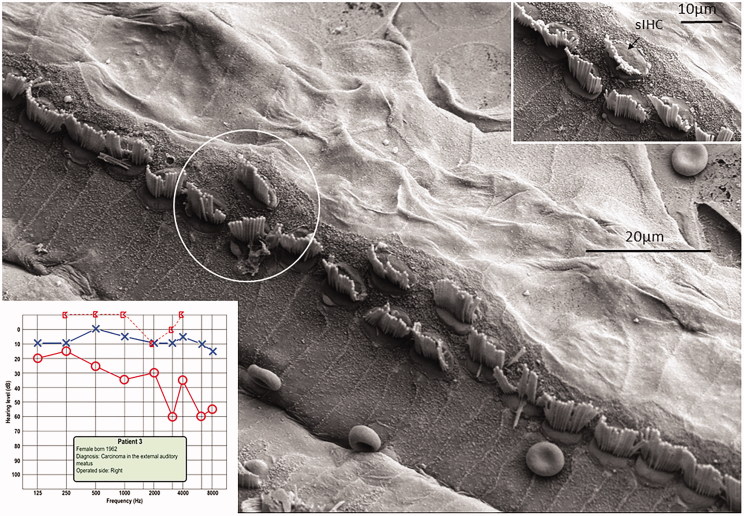
FESEM of the IHC region at the second turn of the human cochlea (patient 3). Sensorineural hearing is normal, with a minor conductive loss due to a carcinoma of the external auditory canal. The IHCs are not well aligned. Several sIHCs are located at the modiolar side (arrows). Microvilli-rich border cells surround the sIHCs (inset). The surface of the inner sulcus is smooth. Some IHC stereocilia are not arranged in straight lines parallel to the long axis of the cochlear duct (encircled).

**Figure 8.**
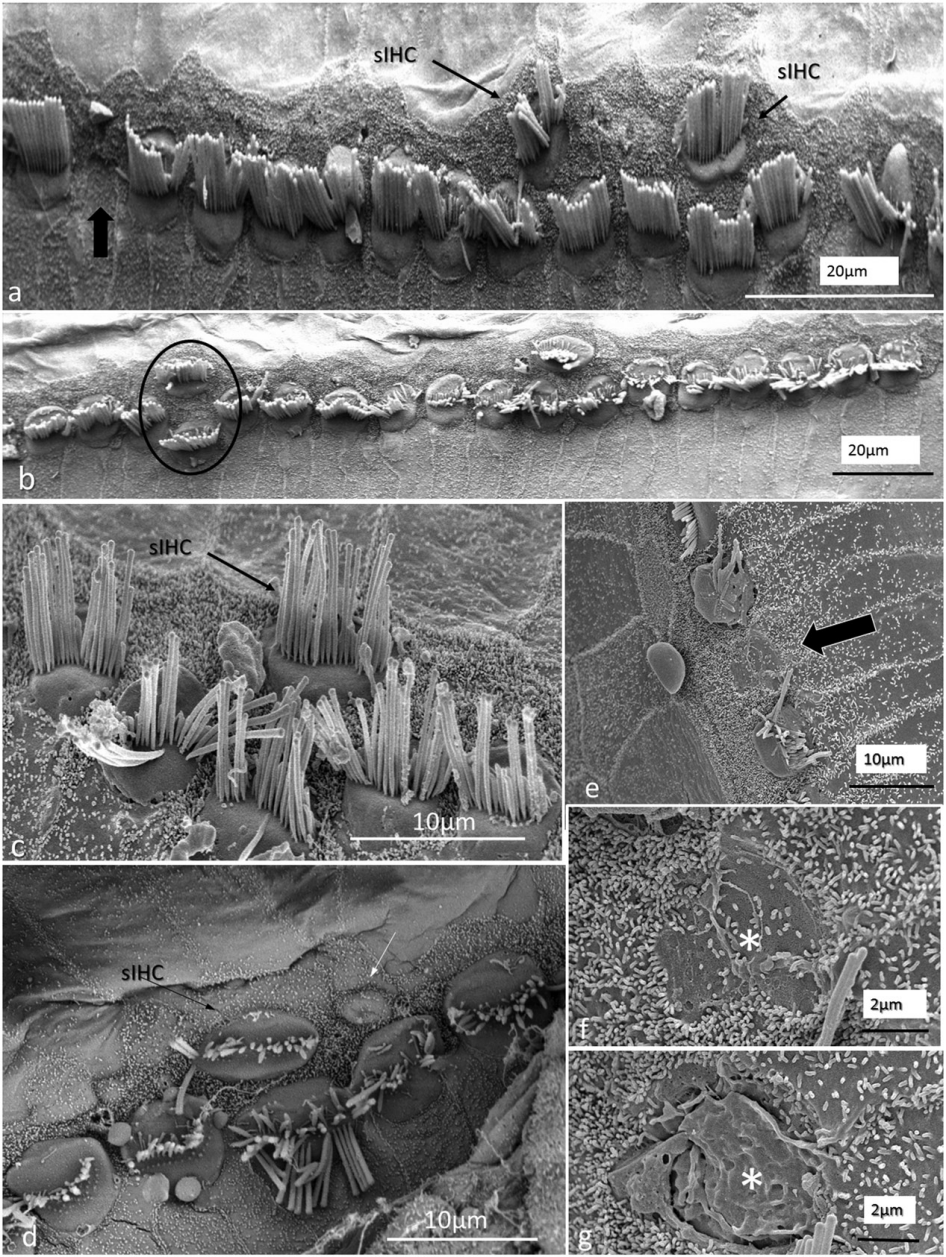
FESEM of the IHC region at the second turn of the human cochlea (patient 3). Image a: sIHCs are located at the modiolar side. The stereocilia bundle of the left sIHC is not well aligned and forms an angle of almost 90° with the IHC row. The surface of the bordering cells is rich in microvilli, whereas the surface of the inner sulcus cells is smooth. One IHC is apparently missing (filled arrow). Image b: At one site (encircled), two parallel hair cells are present. Image c: A sIHC in the upper region of the second turn. The lengths of the longest stereocilia vary. Image d: The IHC region of the lower second turn. A sIHC can be observed. A bulging border cell is present on the right of the sIHC. Image e: Three degenerated IHCs are present in this region. The center cell (arrow) is replaced with a supporting cell. Image f: Higher magnification of center cell (asterisk) shown in Image e. Image g: The lost IHC is replaced with a cell process from the adjacent supporting cell.

**Figure 9.**
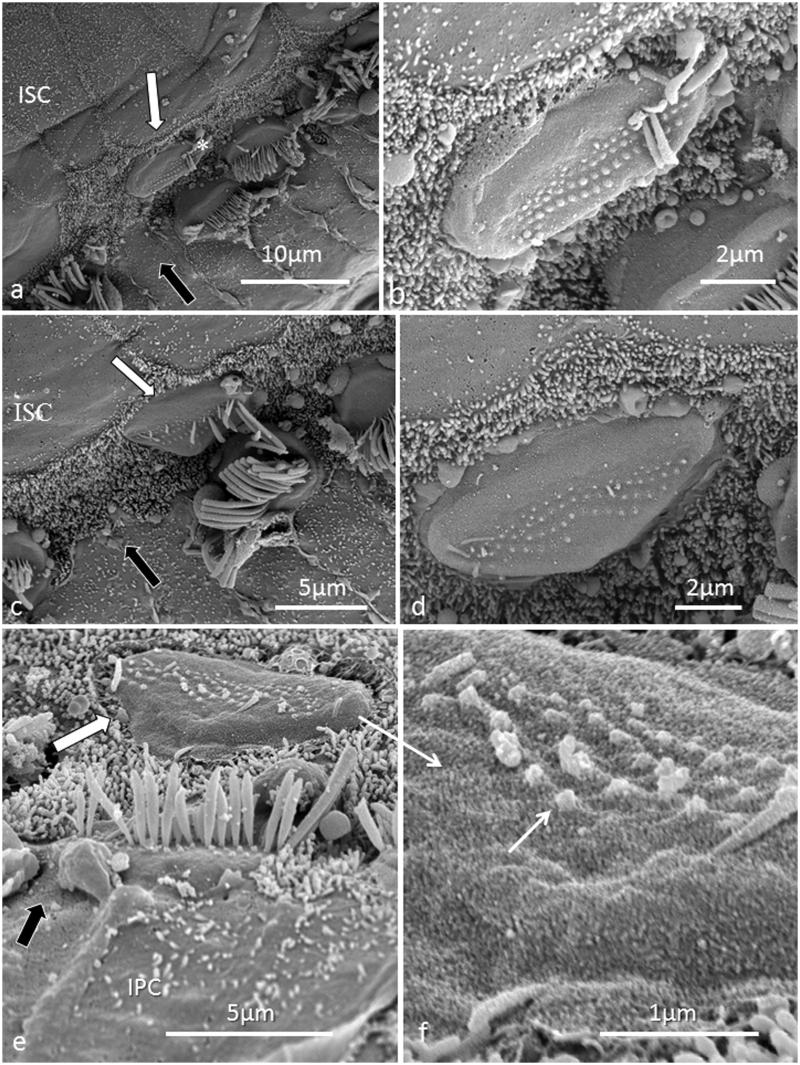
SEM images of sIHCs. Image a: A sIHC is located at the modiolar side of the IHCs near a ‘gap’ with a missing IHC. Image b: An image at higher magnification of the cell shown in Image a. The sIHC stereocilia are rudimentary and stub-like. Image c: A sIHC whose cell surface shows several ciliary stubs and several longer cilia. Image d: Another sIHC near a ‘gap’ with a missing IHC. The surface exhibits several stereocilia stubs. Image e: A sIHC (white arrow) is magnified in Image f. A gap with missing IHCs is marked with a dark arrow.

**Figure 10.**
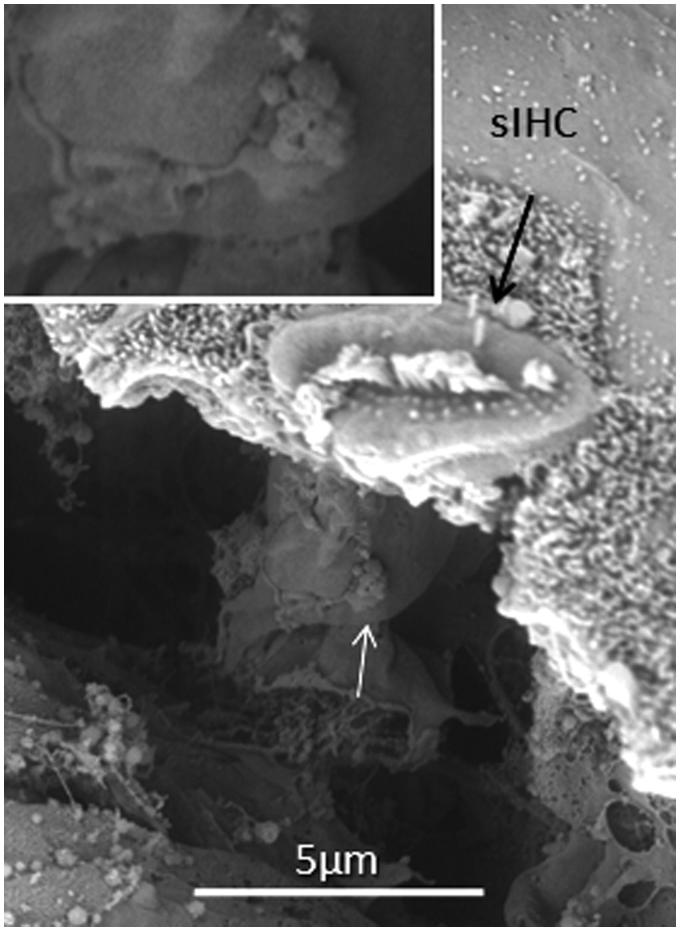
FESEM of a human sIHC at a place where the reticular lamina was ruptured. A synaptic terminal is seen on the lateral wall the cell (arrow).

All specimens exhibited sIHCs in all turns (Figures 3, 4, 5, and 7–9) which were consistently located on the modiolus side of the IHC row. The sIHCs had mostly normal IHC morphology, with well-developed cilia. Their surface plate was ovoid or slightly angular, with no microvilli. Some sIHCs had rudimentary cilia with short stubs. Supernumerary IHCs often showed a shifted cell polarity at deviating angles up to 45° from normal (Figure 8(a)). In patient 1 there were 42 extra IHCs, and 38% were related to a ‘gap’ in the IHC row (±6 IHCs). The ‘gaps’ were observed in all cochlear turns and commonly associated with sIHCs. A few sIHCs had cilia with short stubs, suggesting that they were non-functional. Such stubs were also observed on IHCs, portentous that they were instigated by trauma, such as drilling before fixation. However, similar stubs were observed when drilling was performed after fixation (9). At one place the reticular lamina had ruptured near a sIHC, and it was possible to visualize its lateral surface and find that it was innervated (Figure 10).

## Discussion

Mammalian hair cells are believed to be terminally differentiated with no capacity to replenish, and their loss would inexorably produce permanent functional impairment, unless there is substantial redundancy. The doctrine that these cells are not renewed is based on animal experiments and the clinical experience that patients with sensorineural hearing loss do not regain function. Regeneration of hair cells in the vestibular system of mammals has been reported after gentamicin-induced hair cell loss (23–26). Similar findings were observed in the human utricle (25) and vestibular hair cells following aminoglycoside-induced hair cell loss (27). Immature hair bundles were present in epithelia harvested from patients >60 years of age, suggesting that supporting cells in the human vestibular sensory epithelium may respond to damage by enhancing hair cell regeneration. The authors concluded that spontaneous hair cell regeneration is retained throughout life in human vestibular tissue. A similar activity in the human cochlea has never been verified.

### A small resource of auditory receptors

Human hearing relies on a relatively small number of mechanoreceptor cells, and each cochlea contains around 3,400 IHCs and 12,000 OHCs (22). IHCs are the principal receptors that convert mechanical vibrations into afferent signals in the auditory nerve. Currently, there are joint efforts to explore a means to re-establish the pool of degenerated hair cells. Techniques including the use of stem cells, gene transfer, or induction of potential supporting cells to undergo trans-differentiation or mitotic proliferation have been envisaged (21,28–38). In animal experiments, with p27kip gene disruption, Math1-overexpression, down-regulation of Notch signaling, retinoblastoma gene-deletion, enhancer of Hes1, Hes5, new hair cells may arise with ectopic or supernumerary hair cells (20,21,28–31,38–45). A relevant query is therefore if sIHCs found in the human cochlea reflect ongoing regeneration in man.

### ‘Humans are not simply big mice’

The present study allowed the analysis of the fine structure of well-preserved cochleae from individuals with normal sensory-neural function. Almost 2% of the IHCs were extra IHCs, and they were often related to a ‘one-cell-gap’ in the IHC row and were seen in all cochlear turns. Furthermore, sIHCs were innervated, suggesting that they are functional. Human hair cells’ less rigorous alignment, double IHCs, and irregular mosaic cell pattern are surprising. Human mechano-electric transduction and auditory processing should rely on strictly organized receptor cells along the cochlear axis. This might reflect a modification in the biology of the OC. The limited receptor pool would have an imposing longevity unless there is cell renewal. Consequently, a low-grade cell replacement could explain the negligible loss of IHCs in individuals reaching middle age. If sIHCs reflect a renewal of 1 in every 25 IHCs, the regeneration rate would be considerable. However, in a human transmission electron microscopy (TEM) study of an individual with hearing loss due to chronic noise exposure, there was no hard evidence of IHC regeneration in the damaged 4 kHz area (46). Nonetheless, a certain regenerative capacity cannot be ruled out, since the exposure could also waste support cells. Borg and Viberg (18) studied sIHCs in noise-exposed rabbits and found no difference in unexposed ears (11 sIHCs per cochlea). Maximum numbers were seen at the apex and at 6–9 mm from the apex. The cuticle plate was slightly larger than in ordinary IHCs and had fewer damaged cilia than ordinary IHCs after short-term, high-level noise exposure. The sIHCs appeared less susceptible to noise trauma than ordinary IHCs. There was no evidence of generation of sIHC in the region of hair cell damage.

Microvilli are common on the surface during hair cell development. Supernumerary IHC had no microvilli. Furthermore, during ciliogenesis small cilia buds develop well-aligned stereocilia on the cuticular plate with a subsequent staircase pattern (47–49). Such formation was not observed in the adult human cochlea. Taken together, it cannot be settled if the sIHC represent renewed or redundant accessory IHCs. Further molecular studies are needed to verify if the regenerative capacity of the human auditory periphery might have been underestimated.

## References

[1] Retzius G. Das Gehororgan der Wirbelthiere. Stockholm: Samson and Wallin; 1884.

[2] Lim DJ, Lane WC. Three-dimensional observation of the inner ear with the scanning electron microscope. Trans Am Acad Ophthalmol Otolaryngol. 1969;73:842–72. 4901876

[3] Bredberg G, Lindeman HH, Ades HW, West R, Engstrom H. Scanning electron microscopy of the organ of Corti. Science. 1970;170:861–3. 5482578 10.1126/science.170.3960.861

[4] Furness DN, Hackney CM. High-resolution scanning-electron microscopy of stereocilia using the osmium-thiocarbohydrazide coating technique. Hear Res. 1986;21:243–9. 3522517 10.1016/0378-5955(86)90222-4

[5] Hackney CM, Furness DN, Sayers DL. Stereociliary cross-links between adjacent inner hair cells. Hear Res. 1988;34:207–11. 3170364 10.1016/0378-5955(88)90109-8

[6] Hunter-Duvar IM. Hearing and hair cells. Can J Otolaryngol. 1975;4:152–60. 805644

[7] Hoshino T. Contact between the tectorial membrane and the cochlear sensory hairs in the human and the monkey. Arch Otorhinolaryngol. 1977;217:53–60. 409384 10.1007/BF00453890

[8] Kawabata I, Nomura Y. Extra internal hair cells. A scanning electron microscopic study. Acta Otolaryngol. 1978;85:342–8. 665207 10.3109/00016487809121462

[9] Wright A. Scanning electron microscopy of the human cochlea–postmortem autolysis artefacts. Arch Otorhinolaryngol. 1980;228:1–6. 6781458 10.1007/BF00455888

[10] Wright A. Scanning electron microscopy of the human cochlea–the organ of Corti. Arch Otorhinolaryngol. 1981;230:11–19. 7213191 10.1007/BF00665375

[11] Wright A. Dimensions of the cochlear stereocilia in man and the guinea pig. Hear Res. 1984;13:89–98. 6706866 10.1016/0378-5955(84)90099-6

[12] Engstrom B. Stereocilia of sensory cells in normal and hearing impaired ears. A morphological, physiological and behavioural study. Scand Audiol Suppl. 1983;19:1–34. 6420877

[13] Gleeson MJ. A scanning electron microscopy study of post mortem autolytic changes in the human and rat cochleas. Acta Otolaryngol. 1985;100:419–28. 4082980 10.3109/00016488509126566

[14] Osborne MP, Comis SD, Johnson AP, Jeffries DR. Post-mortem changes in hair bundles of the guinea pig and human cochlea studied by high-resolution scanning microscopy. Acta Otolaryngol. 1989;108:217–26. 2816336 10.3109/00016488909125521

[15] Comis SD, Osborne MP, O'Connell J, Johnson AP. The importance of early fixation in preservation of human cochlear and vestibular sensory hair bundles. Acta Otolaryngol. 1990;109:361–8. 2113759 10.3109/00016489009125156

[16] Glueckert R, Pfaller K, Kinnefors A, Schrott-Fischer A, Rask-Andersen H. High resolution scanning electron microscopy of the human organ of Corti. A study using freshly fixed surgical specimens. Hear Res. 2005;199:40–56. 15574299 10.1016/j.heares.2004.05.006

[17] Rask-Andersen H, Liu W, Erixon E, Kinnefors A, Pfaller K, Schrott-Fischer A, et al Human cochlea: anatomical characteristics and their relevance for cochlear implantation. Anat Rec (Hoboken). 2012;295:1791–811. 23044521 10.1002/ar.22599

[18] Borg E, Viberg A. Extra inner hair cells: prevalence and noise susceptibility. Hear Res. 1995;83:175–82. 7607983 10.1016/0378-5955(94)00200-a

[19] Borg E, Viberg A. Extra inner hair cells in the developing rabbit cochlea. Hear Res. 2002;172:10–13. 12361862 10.1016/s0378-5955(02)00284-8

[20] Abdouh A, Despres G, Romand R. Histochemical and scanning electron microscopic studies of supernumerary hair cells in embryonic rat cochlea in vitro. Brain Res. 1994;660:181–91. 7820686 10.1016/0006-8993(94)91288-2

[21] Lowenheim H, Furness DN, Kil J, Zinn C, Gultig K, Fero ML, et al Gene disruption of p27(Kip1) allows cell proliferation in the postnatal and adult organ of corti. Proc Natl Acad Sci USA. 1999;96:4084–8. 10097167 10.1073/pnas.96.7.4084 PMC22424

[22] Wright A, Davis A, Bredberg G, Ulehlova L, Spencer H, Bock G, et al Hair cell distributions in the normal human cochlea. A report of a European working group. Acta Otolaryngol Suppl. 1987;436:15–24. 3478958 10.3109/00016488709124972

[23] Forge A, Li L, Corwin JT, Nevill G. Ultrastructural evidence for hair cell regeneration in the mammalian inner ear. Science. 1993;259:1616–19. 8456284 10.1126/science.8456284

[24] Forge A, Li L, Nevill G. Hair cell recovery in the vestibular sensory epithelia of mature guinea pigs. J Comp Neurol. 1998;397:69–88. 9671280

[25] Warchol ME, Lambert PR, Goldstein BJ, Forge A, Corwin JT. Regenerative proliferation in inner ear sensory epithelia from adult guinea pigs and humans. Science. 1993;259:1619–22. 8456285 10.1126/science.8456285

[26] Kawamoto K, Izumikawa M, Beyer LA, Atkin GM, Raphael Y. Spontaneous hair cell regeneration in the mouse utricle following gentamicin ototoxicity. Hear Res. 2009;247:17–26. 18809482 10.1016/j.heares.2008.08.010 PMC2905733

[27] Taylor RR, Jagger DJ, Saeed SR, Axon P, Donnelly N, Tysome J, et al Characterizing human vestibular sensory epithelia for experimental studies: new hair bundles on old tissue and implications for therapeutic interventions in ageing. Neurobiol Aging. 2015;36:2068–84. 25818177 10.1016/j.neurobiolaging.2015.02.013 PMC4436436

[28] Zheng JL, Shou J, Guillemot F, Kageyama R, Gao WQ. Hes1 is a negative regulator of inner ear hair cell differentiation. Development. 2000;127:4551–60. 11023859 10.1242/dev.127.21.4551

[29] Zine A, Aubert A, Qiu J, Therianos S, Guillemot F, Kageyama R, et al Hes1 and Hes5 activities are required for the normal development of the hair cells in the mammalian inner ear. J Neurosci. 2001;21:4712–20. 11425898 10.1523/JNEUROSCI.21-13-04712.2001 PMC6762342

[30] Sage C, Huang M, Karimi K, Gutierrez G, Vollrath MA, Zhang DS, et al Proliferation of functional hair cells in vivo in the absence of the retinoblastoma protein. Science. 2005;307:1114–18. 15653467 10.1126/science.1106642

[31] Mantela J, Jiang Z, Ylikoski J, Fritzsch B, Zacksenhaus E, Pirvola U. The retinoblastoma gene pathway regulates the postmitotic state of hair cells of the mouse inner ear. Development. 2005;132:2377–88. 15843406 10.1242/dev.01834 PMC1242168

[32] White PM, Doetzlhofer A, Lee YS, Groves AK, Segil N. Mammalian cochlear supporting cells can divide and trans-differentiate into hair cells. Nature. 2006;441:984–7. 16791196 10.1038/nature04849

[33] Savary E, Hugnot JP, Chassigneux Y, Travo C, Duperray C, Van De Water T, et al Distinct population of hair cell progenitors can be isolated from the postnatal mouse cochlea using side population analysis. Stem Cells. 2007;25:332–9. 17038670 10.1634/stemcells.2006-0303

[34] Shi F, Hu L, Edge AS. Generation of hair cells in neonatal mice by beta-catenin overexpression in Lgr5-positive cochlear progenitors. Proc Natl Acad Sci USA. 2013;110:13851–6. 23918377 10.1073/pnas.1219952110 PMC3752268

[35] Bramhall NF, Shi F, Arnold K, Hochedlinger K, Edge AS. Lgr5-positive supporting cells generate new hair cells in the postnatal cochlea. Stem Cell Reports. 2014;2:311–22. 24672754 10.1016/j.stemcr.2014.01.008 PMC3964281

[36] Li W, Wu J, Yang J, Sun S, Chai R, Chen ZY, et al Notch inhibition induces mitotically generated hair cells in mammalian cochleae via activating the Wnt pathway. Proc Natl Acad Sci USA. 2015;112:166–71. 25535395 10.1073/pnas.1415901112 PMC4291673

[37] Fujioka M, Okano H, Edge AS. Manipulating cell fate in the cochlea: a feasible therapy for hearing loss. Trends Neurosci. 2015;38:139–44. 25593106 10.1016/j.tins.2014.12.004 PMC4352405

[38] Lin V, Golub JS, Nguyen TB, Hume CR, Oesterle EC, Stone JS. Inhibition of Notch activity promotes nonmitotic regeneration of hair cells in the adult mouse utricles. J Neurosci. 2011;31:15329–39. 22031879 10.1523/JNEUROSCI.2057-11.2011 PMC3235543

[39] Doetzlhofer A, Basch ML, Ohyama T, Gessler M, Groves AK, Segil N. Hey2 regulation by FGF provides a Notch-independent mechanism for maintaining pillar cell fate in the organ of Corti. Dev Cell. 2009;16:58–69. 19154718 10.1016/j.devcel.2008.11.008 PMC2696015

[40] Korrapati S, Roux I, Glowatzki E, Doetzlhofer A. Notch signaling limits supporting cell plasticity in the hair cell-damaged early postnatal murine cochlea. PLoS One. 2013;8:e73276. 24023676 10.1371/journal.pone.0073276 PMC3758270

[41] Mizutari K, Fujioka M, Hosoya M, Bramhall N, Okano HJ, Okano H, et al Notch inhibition induces cochlear hair cell regeneration and recovery of hearing after acoustic trauma. Neuron. 2013;77:58–69. 23312516 10.1016/j.neuron.2012.10.032 PMC3573859

[42] Slowik AD, Bermingham-McDonogh O. Hair cell generation by notch inhibition in the adult mammalian cristae. J Assoc Res Otolaryngol. 2013;14:813–28. 23989618 10.1007/s10162-013-0414-z PMC3825023

[43] Zheng JL, Gao WQ. Overexpression of Math1 induces robust production of extra hair cells in postnatal rat inner ears. Nat Neurosci. 2000;3:580–6. 10816314 10.1038/75753

[44] Kawamoto K, Ishimoto S, Minoda R, Brough DE, Raphael Y. Math1 gene transfer generates new cochlear hair cells in mature guinea pigs in vivo. J Neurosci. 2003;23:4395–400. 12805278 10.1523/JNEUROSCI.23-11-04395.2003 PMC6740812

[45] Lefebvr PP, Malgrange B, Thiry M, Breuskin I, Van De Water TR, Moonen G. Supernumerary outer hair cells arise external to the last row of sensory cells in the organ of corti. Acta Otolaryngol. 2001;121:164–8. 11349770 10.1080/000164801300043325

[46] Rask-Andersen H, Ekvall L, Scholtz A, Schrott-Fischer A. Structural/audiometric correlations in a human inner ear with noise-induced hearing loss. Hear Res. 2000;141:129–39. 10713501 10.1016/s0378-5955(99)00216-6

[47] Sanchez-Fernandez JM, Rivera-Pomar JM. Ciliogenesis in human vestibular epithelia. A scanning electron microscopic study. Acta Otolaryngol. 1985;99:405–10. 3874520 10.3109/00016488509108931

[48] Cotanche DA. Regeneration of hair cell stereociliary bundles in the chick cochlea following severe acoustic trauma. Hear Res. 1987;30:181–95. 3680064 10.1016/0378-5955(87)90135-3

[49] Ryals BM, Rubel EW. Hair cell regeneration after acoustic trauma in adult Coturnix quail. Science. 1988;240:1774–6. 3381101 10.1126/science.3381101

